# Whose the Error: God or Man?

**Published:** 1885-12

**Authors:** S. A. Jones


### WHOSE THE ERROR: GOD OR MAN?

While two national societies are "revising" our materia medica, a single physician in far-off Arkansas is interrogating Nature. *His* employment I deem far the worthier; but, alas! he is committing a heinous offense, for he is adding yet another "remedy" to a materia medica that is already "too large!"

Isn't it a pity that the Almighty should have invested each separate plant with its special virtue, and have made so many plants, and thereby have occasioned us so much trouble in mastering our materia medica? If the whole universe contained only ten "remedies," how much nicer would it be, and how compact and how handy our pocket-case would be, and how much simpler the study of materia medica would be! Really, hasn't the Omniscient made a mistake? It looks as if two national societies (when their "revising" job is done) will soon answer this pregnant query in the affirmative. But this is only *my* opinion, and what am *I* compared with two national societies, two editors, and six "consultative" appendages?

Of late years the trend of "our school" is hardly a matter of jubilation to the thoughtful observer. The leaven of agnosticism has found its way into our societies, brought thither by the *Zeitgeist*, and the wily Father of Lies glosses it by calling it "Science," and many are deceived, and the end thereof is—your know *where!* Meanwhile, in far-off Arkansas, one simple-hearted physician makes an old-fashioned "proving," showing that Hahnemann's life-teachings are not *yet* obsolete. Twelve righteous men would have saved Sodom, and there may be twelve left to make old-fashioned "provings" in these United States, and we may be saved—who knows?

Of course, if this Arkansas physician had only made his "proving" under the direction of some materia medica "revising" society, and not according to the old-fashioned Hahnemannian method, his results would be "scientific" and *a la mode;* as it is, his "proving" must enter the lists and win its spurs despite all our "revising" societies. This it can do, and just this every true thing will do, maugre all manner of societies known to men. It is a pity that it is so, for the fact hints that truth *was* even before "revising" societies were.—*Dr. S. A. Jones in Bulletin of Homœopathic News.*

